# Rastreamento do câncer do colo do útero no Brasil: análise da cobertura a partir do Sistema de Informação do Câncer

**DOI:** 10.1590/0102-311XPT152224

**Published:** 2025-09-01

**Authors:** Caroline Madalena Ribeiro, Itamar Bento Claro, Jeane Glaucia Tomazelli, Maria Beatriz Kneipp Dias

**Affiliations:** 1 Instituto Nacional de Câncer, Rio de Janeiro, Brasil.

**Keywords:** Neoplasias do Colo do Útero, Programas de Rastreamento, Políticas, Planejamento e Administração em Saúde, Uterine Cervical Neoplasms, Mass Screening, Health Policy, Planning and Management, Neoplasias del Cuello Uterino, Tamizaje Masivo, Políticas, Planificación y Administración en Salud

## Abstract

O objetivo deste estudo foi analisar a cobertura de rastreamento do câncer do colo do útero no Brasil e Unidades da Federação (UF), utilizando dados secundários do Sistema de Informação do Câncer (SISCAN), comparar com o indicador de razão de exames na população feminina, utilizado classicamente como *proxy* da cobertura e estimar a cobertura possível, caso as diretrizes nacionais preconizadas pelo Ministério da Saúde fossem seguidas adequadamente pelos profissionais de saúde. Foram selecionados exames citopatológicos realizados entre 2021 e 2023, em mulheres até 64 anos de idade, registrados no SISCAN e no Sistema de Informações Ambulatoriais do SUS (SIA/SUS). Indicadores de cobertura e razão foram calculados por UF de residência. Estimou-se a cobertura possível de rastreamento somando todos os exames realizados no período em mulheres até 64 anos e dividindo-se pela população-alvo (25 a 64 anos). A cobertura de rastreamento estimada para o Brasil foi de 35,6%, inferior aos valores do indicador de razão (47,4 e 47,8 por 100 mulheres pelo SISCAN e SIA/SUS, respectivamente). Verificou-se que ao direcionar os exames realizados para faixa etária e periodicidade adequadas, a cobertura poderia atingir 53,9% no Brasil e superar 70% no Espírito Santo, Paraná e Santa Catarina. Concluiu-se que o indicador de razão superestima a cobertura em aproximadamente 35% e que as coberturas de rastreamento estão muito abaixo dos 70% preconizados pela Organização Mundial da Saúde. Entretanto, a sensibilização dos profissionais quanto às diretrizes do Ministério da Saúde podem modificar esse cenário, otimizando recursos e gerando impacto na incidência e mortalidade por câncer do colo do útero.

## Abstract (en)

This study aimed to analyze the coverage of cervical cancer screening in Brazil and its Federative Units (UF, acronym in Portuguese) using secondary data from the Brazilian Cancer Information System (SISCAN, acronym in Portuguese), and to compare it with the index of exam rate in females, classically used as a proxy for coverage, and to estimate the possible coverage, if the national guidelines recommended by the Brazilian Ministry of Health were properly followed by health professionals. Cytopathological tests performed between 2021 and 2023 in women up to 64 years old, registered in SISCAN and in the Outpatient Information System of the SUS (SIA/SUS, acronym in Portuguese), were selected. Coverage and ratio indicators were calculated by UF of residence. The estimated screening coverage was estimated by adding up all the tests performed in the period in women up to 64 years old and dividing by the target population (25-64 years). The estimated screening coverage for Brazil was 35.6%, which is lower than the values of the index of exam rate (47.4 and 47.8 per 100 women according to SISCAN and SIA/SUS, respectively). It was found that by directing the tests to the appropriate age group and periodicity, coverage could reach 53.9% in Brazil and exceed 70% in Espírito Santo, Paraná and Santa Catarina. It was concluded that the rate index overestimates coverage by approximately 35% and that screening coverage is far below the 70% recommended by the World Health Organization. However, the sensitization of professionals regarding the guidelines of the Brazilian Ministry of Health can change this scenario, optimizing resources and generating a positive impact on cervical cancer incidence and mortality.

## Abstract (es)

El objetivo de este estudio fue analizar la cobertura del tamizaje del cáncer de cuello uterino en Brasil y sus Unidades Federativas (UF), utilizando datos secundarios del Sistema de Información sobre el Cáncer (SISCAN), compararla con el indicador de razón de pruebas en la población femenina, utilizado clásicamente como proxy de la cobertura, y estimar la cobertura posible si las directrices nacionales recomendadas por el Ministerio de Salud fueran seguidas adecuadamente por los profesionales. Se seleccionaron pruebas citológicas realizadas entre 2021 y 2023 en mujeres de hasta 64 años, registradas en el SISCAN y en el Sistema de Informaciones Ambulatorias del SUS (SIA/SUS). Los indicadores de cobertura y razón se calcularon por estado federativo de residencia. Se estimó la cobertura posible sumando todas las pruebas realizadas en el período en mujeres de hasta 64 años y dividiéndolas por la población objetivo (25 a 64 años). La cobertura estimada para Brasil fue del 35,6%, inferior a los valores del indicador de razón (47,4 y 47,8 por 100 mujeres según SISCAN y SIA/SUS, respectivamente). Se comprobó que, al orientar las pruebas realizadas hacia el grupo de edad y la periodicidad adecuados, la cobertura podría alcanzar el 53,9% en Brasil y superar el 70% en Espírito Santo, Paraná y Santa Catarina. Se concluyó que el indicador de razón sobreestima la cobertura en aproximadamente un 35% y que las coberturas actuales están muy por debajo del 70% recomendado por la Organización Mundial de la Salud. Sin embargo, la sensibilización de los profesionales sobre las directrices del Ministerio de Salud puede modificar esta situación, optimizando los recursos y generando un impacto en la incidencia y en la mortalidad por cáncer de cuello uterino.

## Introdução

Ainda que sua incidência possa ser reduzida por meio da detecção precoce de lesões pré-malignas, o câncer de colo do útero é o terceiro câncer mais incidente em mulheres no Brasil, com uma taxa bruta de 15,38 casos a cada 100 mil mulheres ^1^.

Ações para reduzir a ocorrência do câncer de colo do útero iniciaram-se de forma mais estruturada na década de 1990, com a oferta do exame citopatológico para uma população-alvo definida e a estruturação de ações de um programa em âmbito nacional, que foi se consolidando ao longo do tempo ^2^.

No Brasil, o rastreamento do câncer do colo do útero é recomendado pelo Ministério da Saúde. Diretrizes nacionais publicadas em 2016 orientam que mulheres de 25 a 64 anos realizem o exame citopatológico a cada três anos, após dois exames anuais consecutivos com resultados normais ^3^.

Para acompanhar as ações e monitorar os indicadores do programa, foi desenvolvido o Sistema de Informação do Câncer de Colo do Útero (SISCOLO), utilizado nacionalmente em diferentes níveis de gestão. Em 2013, o SISCOLO passou por uma importante atualização, com migração para uma plataforma *web* e utilização do Cartão Nacional de Saúde como identificador único do usuário do Sistema Único de Saúde (SUS). Essa nova versão, denominada Sistema de Informação do Câncer (SISCAN), permite identificar as mulheres que realizaram o exame citopatológico no SUS, e não apenas o número de exames ofertados, mas também calcular a cobertura do rastreamento para o câncer de colo do útero na população-alvo, mulheres de 25 a 64 anos, além de acompanhar a ampliação da oferta do exame ^4^.

Os registros dos exames no SISCOLO entre 1999 e 2013 não possuíam identificador único, fragilizando o monitoramento da cobertura do rastreamento e o seguimento adequado das mulheres com exames alterados. A cobertura do rastreamento no SUS tem sido tradicionalmente aferida por meio de um indicador de razão entre exames registrados e mulheres da população-alvo, considerado *proxy* da cobertura ^5^. Uma limitação importante na utilização desse indicador é a possível superestimação dos resultados, devido à baixa adesão às diretrizes, com frequência elevada de exames realizados anualmente.

Estudos realizados em algumas localidades do Brasil indicam baixa adesão às diretrizes de periodicidade do exame, sendo a periodicidade anual muito frequente. Dados de 2006 a 2011 de Minas Gerais revelaram que mais de 50% dos exames foram realizados anualmente, enquanto em estudo mais recente realizado na Bahia, identificou-se que apenas cerca de 10% dos exames foram realizados na periodicidade trienal entre 2017 e 2021 ^6^ ^,^ ^7^. Portanto, utilizar os dados de exames realizados para estimar a cobertura provoca um viés, no sentido de superestimar o número de mulheres examinadas, uma vez que, considera-se que, no período cada exame, deveria representar uma mulher rastreada.

Os dados mais recentes da *Pesquisa Nacional por Amostra de Domicílios* (PNAD), da *Pesquisa Nacional de Saúde* (PNS) e do *Sistema de Vigilância de Fatores de Risco e Proteção para Doenças Crônicas por Inquérito Telefônico* (Vigitel) apontam para coberturas do exame citopatológico acima de 70% na população-alvo (25 a 64 anos) ^8^ ^,^ ^9^. Entretanto, apesar do aumento no acesso aos exames de rastreamento ocorrido nas últimas décadas, os dados das pesquisas nacionais precisam ser considerados com cautela, pois essas coberturas baseadas em informações autorrelatadas podem estar superestimadas, além de incluírem dados de toda a população, inclusive as que realizam exames na saúde suplementar ^9^.

A partir de 2013, com a implantação do SISCAN e a exigência de informação do número do Cartão Nacional de Saúde para registro dos exames, tornou-se possível identificar individualmente as mulheres e realizar o cálculo da cobertura real do rastreamento. Porém, a implantação do sistema ocorreu de forma gradual em todo o país, alcançando 90% dos estabelecimentos que realizam a citologia, em períodos mais recentes ^10^.

No período de transição entre o SISCOLO e o SISCAN, a fonte de informações para o cálculo do indicador de razão mais frequentemente utilizada foi o Sistema de Informações Ambulatoriais do SUS (SIA/SUS), que registra os procedimentos para fins de faturamento ^11^.

A implantação do SISCAN, um sistema de informação com identificador único, possibilita conhecer, de fato, a real cobertura dos exames realizados pelo SUS no país. Por isso, este estudo teve como objetivo analisar a cobertura de rastreamento do câncer do colo do útero no Brasil e Unidades da Federação (UF), utilizando dados secundários do SISCAN, comparar com o indicador de razão de exames na população feminina, utilizado classicamente como *proxy* da cobertura, e estimar a cobertura possível, caso as diretrizes nacionais preconizadas pelo Ministério da Saúde fossem seguidas adequadamente pelos profissionais de saúde.

## Métodos

### Desenho do estudo

Estudo transversal que analisa indicadores relativos à cobertura de rastreamento para o câncer do colo do útero no Brasil e UF no triênio 2021-2023.

### Sujeitos da pesquisa

Foram selecionados todos os exames citopatológicos para o rastreamento do câncer do colo do útero realizados em mulheres de até 64 anos de idade, registrados no SISCAN e SIA/SUS, entre 2021 e 2023.

### Variáveis

Foram selecionadas as variáveis faixa etária (abaixo de 25 anos, 25 a 64 anos), ano do resultado do exame (2021, 2022 e 2023), tipo de exame (rastreamento) e UF de residência.

### Fontes de dados

Os dados referentes aos exames citopatológicos foram obtidos no SISCAN e no SIA/SUS, por meio do tabulador de dados públicos do Departamento de Informática do SUS (DATASUS) ^12^.

O número de mulheres que realizaram o exame citopatológico foi obtido do painel do SISCAN agregado por pacientes, por meio do tabulador de dados públicos do DATASUS ^13^.

Dados referentes à implantação do SISCAN foram retirados de publicação específica sobre o tema. O percentual de implantação refere-se à proporção de serviços de citopatologia com registro de exames citopatológicos no SISCAN em relação aos serviços que registraram o procedimento no sistema de faturamento (SIA/SUS) ^10^.

Os dados referentes à população residente dos estados foram obtidos do *Censo 2022* do Instituto Brasileiro de Geografia e Estatística (IBGE) ^14^.

Os dados referentes à saúde suplementar foram obtidos no tabulador público da Agência Nacional de Saúde Suplementar (ANS) ^15^.

### Métodos quantitativos

Foram calculados os seguintes indicadores: (i) Cobertura de rastreamento para câncer do colo do útero no SUS; (ii) Razão entre exames e a população feminina a cada 100 mulheres; (iii) Razão entre o número de exames registrados no SISCAN e o número de mulheres rastreadas; (iv) Diferença entre o número de exames na população-alvo registrados no SISCAN e o número de mulheres rastreadas; e (v) Cobertura possível.

#### Cobertura de rastreamento para câncer do colo do útero no SUS

$$\frac{Número\;de\;mulheres\;de\;25\;a\;64\;anos\;rastreadas\;entre\;2021\;e\;2023}{População\;feminina\;de\;25\;a\;64\;anos\;usuária\;exclusivamente\;do\;SUS}x\;100$$

Indica a proporção de mulheres da população-alvo (25 a 64 anos) que realizou o exame de rastreamento no período de três anos. Nesse indicador, cada mulher é contabilizada apenas uma vez, mesmo que tenha realizado mais de um exame durante o período.

A população usuária exclusivamente do SUS foi obtida pela subtração da população coberta por planos privados de saúde da população total nessa faixa etária.

A população coberta por planos privados de saúde foi estimada multiplicando-se o percentual de cobertura de saúde suplementar pelo número de mulheres de 25 a 64 anos. Devido à disponibilidade dos dados de cobertura da ANS apenas por grupos etários, utilizou-se o percentual de cobertura na população feminina de 20 a 69 anos, faixa etária mais próxima do estudo.

#### Razão entre exames e a população feminina a cada 100 mulheres

$$\frac{Número\;de\;exames\;realizados\;em\;mulheres\;de\;25\;a\;64\;anos\;entre\;2021\;e\;2023}{População\;feminina\;de\;25\;a\;64\;anos\;usuária\;exclusivamente\;do\;SUS}x\;100$$

Indica a relação entre o número de exames de rastreamento realizados a cada 100 mulheres da população-alvo e o número de usuárias exclusivamente do SUS, no período de três anos. Indicador tradicionalmente utilizado como *proxy* da cobertura, quando os dados individualizados não estavam disponíveis.

Esse indicador foi calculado utilizando duas fontes distintas para o numerador: os registros do SIA/SUS e os registros do SISCAN, possibilitando a comparação entre as fontes e a verificação da magnitude da diferença, principalmente nos estados com baixa implantação do SISCAN (Piauí, Rio de Janeiro e São Paulo).

#### Razão entre o número de exames registrados no SISCAN e o número de mulheres rastreadas

$$\frac{Número\;de\;exames\;registrados\;no\;SISCAN\;em\;mulheres\;de\;25\;a\;64\;anos}{Número\;de\;mulheres\;de\;25\;a\;64\;anos\;examinadas}$$

Estima quantos exames de rastreamento foram realizados para cada mulher examinada no período de três anos.

#### Diferença entre o número de exames na população-alvo registrados no SISCAN e o número de mulheres rastreadas

Número de exames registrados no SISCAN em mulheres de 25 a 64 anos-Número de mulheres rastreadas

Estima o excesso de exames realizados na população-alvo no período de três anos, considerando ser indicado apenas um exame para cada mulher.

#### Cobertura possível

Esse indicador foi calculado para estimar a cobertura possível caso os exames de rastreamento fossem ofertados seguindo as diretrizes nacionais de periodicidade e faixa etária. Primeiro, calculou-se a cobertura possível se todos os exames realizados em mulheres de 25 a 64 anos fossem realizados na periodicidade trienal, utilizando a seguinte fórmula:

$$\frac{Total\;de\;exames\;realizados\;em\;mulheres\;de\;25\;a\;64\;anos\;entre\;2021\;e\;2023}{População\;feminina\;de\;25\;a\;64\;anos\;usuária\;exclusivamente\;do\;SUS}x\;100$$

Posteriormente, calculou-se a cobertura possível se, além da realização de exames na periodicidade trienal, aqueles realizados em mulheres com menos de 25 anos, fora da faixa etária alvo recomendada, também fossem direcionados à população-alvo. Optou-se por não incluir, nesse cálculo, os exames realizados em mulheres com 65 anos ou mais, porque as diretrizes recomendam que mulheres dessa faixa etária que nunca tenham realizado o exame, realizem dois exames com intervalo de um a três anos, e se ambos os exames forem negativos, encerrar o rastreamento ^3^.

$$\frac{Número\;de\;exames\;de\;rastreamento\;realizados\;em\;mulheres\;de\;até\;64\;anos\;de\;idade\;entre\;2021\;e\;2023}{População\;feminina\;de\;25\;a\;64\;anos\;usuária\;exclusivamente\;do\;SUS}x\;100$$

Os indicadores foram calculados por UF de residência, porém nos cálculos para o Brasil, optou-se por excluir a população e os exames realizados nas UFs com implantação do SISCAN, em 2022, inferior a 86% (Piauí - 44,8%, Rio de Janeiro - 76,3% e São Paulo - 52,1%), considerando que sua inclusão subestimaria as estimativas de cobertura pela sub-representatividade dos exames dessas UFs ^10^.

### Aspectos éticos

O projeto do estudo não foi submetido à apreciação de um comitê de ética em pesquisa por utilizar exclusivamente dados secundários, de acesso público e sem a possibilidade de identificação dos indivíduos submetidos aos procedimentos de diagnóstico e tratamento oncológico analisados, sendo, portanto, dispensado de aprovação por parte de comitê de ética em pesquisa, conforme a *Resolução nº 510/2016*, da Comissão Nacional de Ética em Pesquisa (CONEP).

## Resultados

Entre 2021 e 2023, foram realizados no SUS e registrados no SISCAN 17.165.172 exames citopatológicos de rastreamento em mulheres de 25 a 64 anos. Nos estados onde o SISCAN estava amplamente implementado, foram contabilizados 14.288.170 exames realizados em 10.720.808 mulheres. A cobertura de rastreamento no SUS no período analisado foi de 35,6% (Tabela 1).

**Tabela 1:** Número de exames citopatológicos e de mulheres examinadas, população feminina usuária exclusivamente do Sistema Único de Saúde (SUS), cobertura de rastreamento e razão entre exames e mulheres de 25 a 64 anos, segundo Unidades da Federação (UF) de residência. Brasil, 2021 a 2023.

UF de residência	População feminina usuária do SUS	SISCAN			SIA/SUS		
		Exames realizados	Mulheres examinadas	Cobertura (%)	Razão de exames por 100 mulheres	Exames realizados	Razão de exames por 100 mulheres
Brasil *	30.149.313	14.288.170	10.720.808	35,6	47,4	14.419.311	47,8
Rondônia	380.941	131.415	105.092	27,6	34,5	152.752	40,1
Acre	187.001	88.043	66.153	35,4	47,1	111.756	59,8
Amazonas	761.660	492.551	361.895	47,5	64,7	486.626	63,9
Roraima	138.600	46.676	37.629	27,1	33,7	55.852	40,3
Pará	1.806.624	585.603	450.639	24,9	32,4	593.893	32,9
Amapá	161.652	34.271	29.090	18,0	21,2	51.126	31,6
Tocantins	354.116	97.030	78.348	22,1	27,4	114.474	32,3
Maranhão	1.596.910	553.188	415.290	26,0	34,6	494.913	31,0
Piauí	769.580	211.548	166.384	21,6	27,5	335.349	43,6
Ceará	2.022.326	795.286	591.027	29,2	39,3	783.121	38,7
Rio Grande do Norte	738.302	282.161	212.777	28,8	38,2	262.353	35,5
Paraíba	956.396	452.761	320.228	33,5	47,3	410.429	42,9
Pernambuco	2.112.357	965.206	687.199	32,5	45,7	964.518	45,7
Alagoas	737.625	442.578	312.760	42,4	60,0	454.903	61,7
Sergipe	518.340	223.571	168.574	32,5	43,1	233.390	45,0
Bahia	3.449.331	1.416.032	1.072.379	31,1	41,1	1.399.678	40,6
Minas Gerais	4.079.780	2.296.058	1.770.969	43,4	56,3	2.356.760	57,8
Espírito Santo	682.480	491.648	360.524	52,8	72,0	475.884	69,7
Rio de Janeiro	3.027.944	742.440	617.686	20,4	24,5	1.030.210	34,0
São Paulo	6.989.691	1.923.012	1.467.991	21,0	27,5	4.996.228	71,5
Paraná	2.235.208	1.392.547	1.035.593	46,3	62,3	1.439.214	64,4
Santa Catarina	1.576.520	1.009.750	756.527	48,0	64,0	914.448	58,0
Rio Grande do Sul	2.290.366	1.266.835	927.245	40,5	55,3	1.327.608	58,0
Mato Grosso do Sul	544.523	256.237	197.432	36,3	47,1	365.822	67,2
Mato Grosso	764.844	354.852	267.471	35,0	46,4	342.058	44,7
Goiás	1.543.599	460.692	368.401	23,9	29,8	458.290	29,7
Distrito Federal	509.813	153.179	127.566	25,0	30,0	169.443	33,2

SIA/SUS: Sistema de Informações Ambulatoriais do SUS; SISCAN: Sistema de Informações do Câncer.

Fonte: SISCAN ^12^ ^,^ ^13^, SIA/SUS ^10^, Agência Nacional de Saúde Suplementar ^15^ e Instituto Brasileiro de Geografia e Estatística ^14^.

* Excluindo os dados de Piauí, Rio de Janeiro e São Paulo (estados com baixa implantação do SISCAN).

As maiores coberturas de rastreamento foram identificadas no Espírito Santo (52,8%), Santa Catarina (48%), Amazonas (47,5%) e Paraná (46,3%). Excluindo os estados com baixa implantação do SISCAN (Piauí, Rio de Janeiro e São Paulo), cujos resultados estão subestimados, as menores coberturas foram identificadas no Amapá (18%), Tocantins (22,1%) e Pará (24,9%).

O indicador de razão entre exames e a população-alvo foi de 47,4/100 mulheres, utilizando o SISCAN como fonte, e 47,8/100 mulheres, quando utilizado o SIA/SUS como fonte de dados. Os valores foram semelhantes ao usar as duas fontes de dados, exceto nos estados com baixa implantação do SISCAN, Piauí e São Paulo, e no Mato Grosso do Sul. O indicador de razão superestimou os valores de cobertura em cerca de 34% (Tabela 1).

As maiores diferenças entre a cobertura e o indicador de razão calculado com dados do SIA/SUS ocorreram no Mato Grosso do Sul (cobertura 36,3% e razão 67,2/100), Acre (cobertura 35,4% e razão 59,8/100) e Amapá (cobertura 18% e razão 31,6/100) (Tabela 1).

Ao comparar o número de exames de rastreamento realizados e o número de mulheres examinadas, observa-se que em média cada mulher realizou 1,3 exame no período de três anos, resultando em um excesso de aproximadamente 3,5 milhões de exames no período, entre as mulheres de 25 a 64 anos (Tabela 2).

**Tabela 2:** Excesso de exames realizados e estimativa de cobertura possível considerando a periodicidade trienal. Brasil e Unidades da Federação (UF), 2021 a 2023.

UF de residência	Exames realizados	Mulheres examinadas	Relação exames/mulheres	Excesso de exames	População usuária do SUS	Cobertura possível (%)
Brasil *	14.288.170	10.720.808	1,3	3.567.362	30.149.313	47,4
Rondônia	131.415	105.092	1,3	26.323	380.941	34,5
Acre	88.043	66.153	1,3	21.890	187.001	47,1
Amazonas	492.551	361.895	1,4	130.656	761.660	64,7
Roraima	46.676	37.629	1,2	9.047	138.600	33,7
Pará	585.603	450.639	1,3	134.964	1.806.624	32,4
Amapá	34.271	29.090	1,2	5.181	161.652	21,2
Tocantins	97.030	78.348	1,2	18.682	354.116	27,4
Maranhão	553.188	415.290	1,3	137.898	1.596.910	34,6
Piauí	211.548	166.384	1,3	45.164	769.580	27,5
Ceará	795.286	591.027	1,3	204.259	2.022.326	39,3
Rio Grande do Norte	282.161	212.777	1,3	69.384	738.302	38,2
Paraíba	452.761	320.228	1,4	132.533	956.396	47,3
Pernambuco	965.206	687.199	1,4	278.007	2.112.357	45,7
Alagoas	442.578	312.760	1,4	129.818	737.625	60,0
Sergipe	223.571	168.574	1,3	54.997	518.340	43,1
Bahia	1.416.032	1.072.379	1,3	343.653	3.449.331	41,1
Minas Gerais	2.296.058	1.770.969	1,3	525.089	4.079.780	56,3
Espírito Santo	491.648	360.524	1,4	131.124	682.480	72,0
Rio de Janeiro	742.440	617.686	1,2	124.754	3.027.944	24,5
São Paulo	1.923.012	1.467.991	1,3	455.021	6.989.691	27,5
Paraná	1.392.547	1.035.593	1,3	356.954	2.235.208	62,3
Santa Catarina	1.009.750	756.527	1,3	253.223	1.576.520	64,0
Rio Grande do Sul	1.266.835	927.245	1,4	339.590	2.290.366	55,3
Mato Grosso do Sul	256.237	197.432	1,3	58.805	544.523	47,1
Mato Grosso	354.852	267.471	1,3	87.381	764.844	46,4
Goiás	460.692	368.401	1,3	92.291	1.543.599	29,8
Distrito Federal	153.179	127.566	1,2	25.613	509.813	30,0

SISCAN: Sistema de Informações do Câncer; SUS: Sistema Único de Saúde.

Fonte: SISCAN ^12^ ^,^ ^13^.

* Excluindo os dados de Piauí, Rio de Janeiro e São Paulo (estados com baixa implantação do SISCAN).

Se os exames de rastreamento realizados fora da periodicidade recomendada fossem direcionados para mulheres nunca rastreadas ou em atraso, as coberturas aumentariam em todos os estados. No Brasil, a cobertura passaria de 35,6% para 47,4%, em Alagoas, passaria de 42,4% para 60%, e no Amazonas, de 47,5% para 64,7%. O Espírito Santo, que apresentou a maior cobertura (52,8%), atingiria 72% de cobertura, se realizasse todos os exames na periodicidade correta (Tabela 2).

Mesmo se os estados com baixa implantação do SISCAN fossem incluídos no cálculo da cobertura nacional, ao considerar que todos os exames registrados fossem realizados na periodicidade correta, já seria possível atingir cobertura superior (42%) àquela encontrada nesse período, quando considerados apenas os estados com boa implantação do sistema (35,6%).

No período analisado, 1,9 milhão de exames foram realizados em mulheres com menos de 25 anos. Se esse quantitativo também fosse direcionado à população-alvo, as coberturas possíveis poderiam atingir 80,6% no Espírito Santo, 73,9% no Amazonas, 72,3% em Santa Catarina e 70,6% no Paraná. No Brasil, a cobertura passaria para 53,9% (Tabela 3).

**Tabela 3:** Exames realizados em mulheres abaixo da faixa etária alvo (< 25 anos) e cobertura possível na população-alvo.

UF de residência	Exames realizados (25-64 anos)	População feminina usuária do SUS (25-64 anos)	Exames realizados em menores de 25 anos	Cobertura possível (%)
Brasil *	14.288.170	30.149.313	1.950.421	53,9
Rondônia	131.415	380.941	20.717	39,9
Acre	88.043	187.001	15.127	55,2
Amazonas	492.551	761.660	70.328	73,9
Roraima	46.676	138.600	8.411	39,7
Pará	585.603	1.806.624	95.389	37,7
Amapá	34.271	161.652	6.385	25,2
Tocantins	97.030	354.116	13.490	31,2
Maranhão	553.188	1.596.910	85.825	40,0
Piauí	211.548	769.580	28.138	31,1
Ceará	795.286	2.022.326	115.880	45,1
Rio Grande do Norte	282.161	738.302	44.897	44,3
Paraíba	452.761	956.396	64.850	54,1
Pernambuco	965.206	2.112.357	155.311	53,0
Alagoas	442.578	737.625	67.735	69,2
Sergipe	223.571	518.340	42.351	51,3
Bahia	1.416.032	3.449.331	217.335	47,4
Minas Gerais	2.296.058	4.079.780	225.630	61,8
Espírito Santo	491.648	682.480	58.542	80,6
Rio de Janeiro	742.440	3.027.944	80.511	27,2
São Paulo	1.923.012	6.989.691	225.797	30,7
Paraná	1.392.547	2.235.208	185.182	70,6
Santa Catarina	1.009.750	1.576.520	130.054	72,3
Rio Grande do Sul	1.266.835	2.290.366	149.721	61,8
Mato Grosso do Sul	256.237	544.523	37.452	53,9
Mato Grosso	354.852	764.844	55.722	53,7
Goiás	460.692	1.543.599	64.164	34,0
Distrito Federal	153.179	509.813	19.923	34,0

SISCAN: Sistema de Informações do Câncer; SUS: Sistema Único de Saúde.

Fonte: SISCAN ^12^ ^,^ ^13^.

* Excluindo os dados de Piauí, Rio de Janeiro e São Paulo (estados com baixa implantação do SISCAN).

## Discussão

Este estudo é o primeiro a apresentar a cobertura de rastreamento do câncer do colo do útero no SUS com base no número de mulheres examinadas, e não no número de exames registrados. A análise dos dados do SISCAN, dez anos após o início de sua implantação, permitiu estimar uma cobertura média de rastreamento para o Brasil, no triênio 2021-2023, de 35,6%. Além disso, identificou-se que o indicador de razão superestima os valores em cerca de 35%, uma vez que a razão entre exames e a população-alvo foi 47,4/100 mulheres, quando utilizado o SISCAN como fonte, e 47,8/100 mulheres quando utilizado o SIA/SUS. Nos diferentes métodos de cálculo, observa-se que todos os estados ainda estão distantes da cobertura adequada para atingir a redução da incidência e da mortalidade por câncer do colo do útero, porém somente o direcionamento adequado da oferta em quatro localidades (Amazonas, Espírito Santo, Paraná e Santa Catarina) já seria suficiente para alcançá-la. Portanto, ações de gestão da oferta do exame citopatológico podem trazer impacto na incidência de lesões precursoras e a longo prazo na incidência de câncer de colo do útero.

A implantação não uniforme do SISCAN nas UFs limitou a análise do estudo, devido à exclusão de três UFs nos indicadores do Brasil. Entretanto, acredita-se que os resultados sejam representativos do Brasil, já que englobam a maior parte dos estados. Outro ponto que pode ser considerado são as situações em que há necessidade de repetição do exame citopatológico em periodicidade inferior a 3 anos, como em exames de primeira vez e em exames insatisfatórios, situações não contempladas na análise, mas que representam uma parcela pequena do universo de exames. Nas situações em que o exame precisa ser repetido pela conduta diagnóstica indicada, como nos resultados de lesões de baixo grau, este não deve ser registrados no SISCAN como exame de rastreamento, e sim de repetição, porém é possível que erros de preenchimento possam ter levado à sua inclusão na análise. 

Em 2020, a Organização Mundial da Saúde (OMS) lançou a estratégia global de eliminação do câncer do colo do útero, que tem como meta reduzir as taxas de incidência da doença em todos os países para menos de quatro casos por 100 mil mulheres. Para alcançar esse objetivo, foram definidos como pilares a prevenção, o rastreamento e o tratamento, com as seguintes metas: vacinar contra o HPV 90% das meninas até os 15 anos de idade; rastrear 70% das mulheres entre 35 e 45 anos pelo menos duas vezes na vida com um teste de alta performance; e tratar 90% das mulheres com lesões precursoras ou câncer invasivo ^16^.

Apesar de uma série de ações e programas relacionados a prevenção e detecção precoce do câncer do colo do útero terem sido implementadas no Brasil desde a década de 1980, com ampliação da oferta de rastreamento e inclusão da cobertura de exame na população-alvo, calculada pela *proxy*, nas metas e indicadores pactuados por estados e municípios, observa-se que as estimativas de cobertura no SUS e oferta de procedimentos ainda estão abaixo do necessário ^17^.

As maiores coberturas foram encontradas nos estados das regiões Sudeste e Sul, corroborando os achados de estudos baseados em dados de exames e inquéritos populacionais ^9^ ^,^ ^18^ ^,^ ^19^ ^,^ ^20^. Na Região Sudeste, não foi possível estimar o verdadeiro cenário de cobertura, uma vez que São Paulo e Rio de Janeiro apresentavam baixa implantação do SISCAN. Analisando os dados de produção de exames no SIA/SUS, é possível supor que o Estado de São Paulo tenha uma das coberturas mais elevadas do país, já que são realizados 71 exames para cada 100 mulheres da população-alvo, valor superior ao do Espírito Santo, que apresentou a maior cobertura entre os estados com implantação adequada do SISCAN.

As baixas coberturas encontradas nos estados da Região Norte merecem atenção especial, por se tratar da região com as maiores taxas de incidência e mortalidade por câncer do colo do útero e não ter acompanhado a queda na mortalidade observada no país ^21^. O acesso ao rastreamento no Norte é uma barreira importante, visto que, mesmo com o direcionamento adequado da oferta atual, a cobertura ainda seria inferior a 40% na maior parte dos estados.

O Estado do Amazonas possui a maior incidência de câncer do colo do útero do país, apresentando a maior cobertura de rastreamento da Região Norte ^1^, bem como a possibilidade de alcançar cobertura de 70% seguindo as recomendações vigentes. Esse achado reforça a necessidade de qualificação dos profissionais da atenção primária à saúde (APS) para a correta indicação do exame, que pode contribuir para a redução da morbimortalidade pela doença com os recursos já existentes hoje.

A situação do Amapá também merece destaque: com a menor cobertura encontrada (18%), apresenta a segunda maior taxa de incidência do país e a estimativa de cobertura possível é de apenas 25% da população-alvo, sendo necessário identificar ações para a ampliação da oferta de exames e cobertura de rastreamento ^1^.

Nos estados das regiões Sul e Sudeste, encontram-se as menores taxas de incidência do país e as maiores coberturas, chamando atenção a situação de Santa Catarina, cuja taxa de incidência estimada é de 17,2 casos por 100 mil mulheres, a maior das regiões Sul e Sudeste, e também a segunda maior cobertura de rastreamento do país. Esse achado inesperado merece atenção para identificar se apesar da maior cobertura há falhas no sistema de saúde que ocasionem a elevada incidência.

Ao analisar indicadores de processo referentes à detecção precoce desse câncer, observa-se desigualdades regionais marcantes, que também podem estar associadas à maior incidência e mortalidade. Dados do relatório anual do Instituto Nacional de Câncer indicam baixa capacidade de identificação de alterações nos exames de rastreamento na Região Nordeste (índice de positividade 2,8%) e altos percentuais de laboratórios de baixa produção anual nas regiões Norte (39,4%), Centro-oeste (37,1%) e Nordeste (36,9%), o que pode comprometer a qualidade dos exames e, consequentemente, o diagnóstico de lesões precursoras e câncer em estágios iniciais. Além disso, nessas regiões, encontram-se os maiores tempos de liberação dos laudos anatomopatológicos das biópsias, bem como maiores tempos entre o diagnóstico e início do tratamento ^22^.

É importante ressaltar que o período do estudo, 2021 a 2023, compreende um período ainda afetado pela pandemia de COVID-19, que provocou redução acentuada do rastreamento do câncer do colo do útero no Brasil. Apesar de 2020 ter sido o ano que sofreu maior impacto, com redução de cerca de 50% nos exames de rastreamento, o patamar pré-pandemia foi alcançado apenas em 2022 e ultrapassado em 2023 ^22^ ^,^ ^23^. No ano de 2021, a realização de exames citopatológicos ainda foi 17% inferior a de 2019, porém em 2023 o número de exames realizados superou em 10% os valores de 2019. Assim, é possível que a cobertura calculada tenha sofrido alguma redução relacionada à pandemia e que nos próximos triênios a cobertura de rastreamento aumente, apenas pelo retorno das ações anteriormente realizadas, mesmo que não sejam adotadas medidas para sua ampliação.

A adesão dos profissionais da APS às diretrizes de rastreamento vigentes no país é uma dificuldade relatada por gestores e profissionais de saúde. Em estudo qualitativo realizado com gestores do SUS, as duas principais barreiras apontadas para a implementação das diretrizes para o câncer do colo do útero foram a pouca tradição organizacional no uso de diretrizes (25%) e a baixa adesão dos profissionais (21,4%) ^24^. Em estudo realizado com profissionais de unidades de saúde da família no Brasil em 2011, embora 68,7% dos médicos e 76,9% dos enfermeiros entrevistados tenham relatado que as recomendações nacionais para rastreamento do câncer do colo do útero eram muito influentes em sua unidade de saúde, 84,9% e 96,6%, respectivamente, relataram realizar o exame anualmente ^25^. Em estudo mais recente com profissionais da APS de um município de Minas Gerais, apenas 45,2% demonstraram conhecimento adequado sobre a faixa etária de rastreamento, e 71,5% sobre a periodicidade ^26^.

Neste estudo, foi identificado um excesso de exames realizados fora da periodicidade e da faixa etária recomendadas. Em média, foram realizados 1,3 exame de rastreamento para cada mulher da população-alvo no período de 2021 a 2023, o que gerou um excesso de mais de 3,5 milhões de exames. Aliado a isso, aproximadamente 2 milhões de exames foram realizados em mulheres com idade inferior a 25 anos, quando não há recomendação de rastreamento. Se esse excesso fosse direcionado para a população que não realizou o exame, seria possível alcançar uma cobertura de quase 54% da população usuária exclusivamente do SUS na faixa etária alvo, considerando os estados com maior percentual de implantação do SISCAN. Possivelmente, a cobertura poderia ser ainda maior, se fossem considerados os exames realizados em pessoas com 65 anos ou mais, que não foram incluídas na análise, devido à possibilidade de indicação de exame de rastreamento para aquelas pessoas que nunca foram rastreadas.

A baixa adesão dos profissionais às diretrizes de periodicidade e faixa etária no Brasil vem sendo relatada em estudos anteriores. Pla et al. ^27^ identificaram que cerca de 20% dos exames realizados no Brasil entre 2008 e 2011 estavam fora da faixa etária alvo, e mais de 90% realizados entre 1 e 2 anos de periodicidade. Estudo com dados de 2006 a 2015 estimou um custo de 147,5 milhões de reais com exames realizados fora da faixa etária alvo nesse período ^28^.

Já em um estudo realizado em Campinas (São Paulo), foi verificado melhora nos indicadores de adesão às diretrizes entre 2010 e 2016, sendo possível observar uma tendência de aumento no intervalo de realização do exame de rastreamento e redução no excesso de exames ^29^. A realização de exames fora da periodicidade e população-alvo é reflexo do rastreamento oportunístico realizado no país, o qual depende da oferta do exame quando a mulher procura e/ou tem acesso à unidade de saúde, além da conduta profissional de recomendar o exame anual ^25^ ^,^ ^26^ ^,^ ^30^.

Outro aspecto relevante é que o rastreamento oportunístico falha na captação da população mais vulnerável e sob maior risco de desenvolver câncer do colo do útero. Isso porque, as mesmas mulheres costumam ser sobre rastreadas, e aquelas que nunca realizaram o exame, as quais são, em geral, as de menor renda e escolaridade, são as mais acometidas pela doença ^31^.

O monitoramento da cobertura de rastreamento, possível a partir da consolidação do SISCAN, em breve passará por um desafio referente ao novo método, com nova periodicidade, que passará de 3 para 5 anos, o que exigirá esforços para coletar e analisar adequadamente os dados do programa, possibilitando melhor planejamento das ações.

Entretanto, a experiência do município de Indaiatuba, onde a mudança do modelo oportunístico apoiado na citologia para o modelo organizado, baseado no teste DNA HPV, mostra excelentes resultados, como a cobertura acima de 70% da população-alvo, antecipação do diagnóstico de câncer em 10 anos e o dobro de diagnósticos de lesões de alto grau, quando comparado ao rastreamento oportunístico com citologia ^31^.

Com a recente aprovação da incorporação do teste DNA HPV oncogênico como método de rastreamento no SUS, condicionada a um programa organizado, é fundamental que profissionais de saúde tenham acesso às diretrizes e sejam sensibilizados quanto à importância de sua adesão para o sucesso do programa ^32^. Ações de capacitação para profissionais e gestores precisam ser consideradas nesse processo, para garantir adesão às recomendações e monitoramento da implementação. Além disso, mecanismos regulatórios devem ser aplicados para reduzir o excesso de exames associado a condutas inadequadas que reduzem a efetividade do programa.
